# Overweight and obesity in Slovak high school students and body composition indicators: a non-randomized cross-sectional study

**DOI:** 10.1186/s12889-016-3508-9

**Published:** 2016-08-17

**Authors:** Bibiana Vadasova, Pavol Cech, Viera Smerecka, Jan Junger, Martin Zvonar, Pavel Ruzbarsky

**Affiliations:** 1 Department of Sport Kinanthropology, Faculty of Sports, University of Prešov, 17th November street No. 13, Prešov, Slovak Republic; 2 Department of Educology of Sports, Faculty of Sports, University of Prešov, 17th November street No. 13, Prešov, Slovak Republic; 3 Department of Kinesiology, Faculty of Sports Studies, Masaryk University, Kamenice 5, Brno, Czech Republic

**Keywords:** Bioimpedance, Classification of body mass index, Body composition, Metabolic disease

## Abstract

**Background:**

Physical development can be considered as an indicator of the overall health status of the youth population. Currently, it appears that the increasing trend of the prevalence of obesity among children and youths has stopped in a number of countries worldwide. Studies point to the fact that adolescence is a critical period for the development of obesity. Body mass index (BMI) seems to be an orientation parameter in the assessment of prevalence of obesity which is not sufficient for more accurate identification of at risk individuals.

The purpose of this study was to evaluate association between BMI percentile zones as health-risk for being overweight and obese and body composition indicators in high-school students from the Prešov (Slovakia) region.

**Methods:**

A non-randomized cross-sectional study in high school students from the Prešov (Slovakia) region was conducted. The research sample consisted of 1014 participants (boys *n* = 466, girls *n* = 549). Body composition was measured using direct segmental multi-frequency bioelectrical impedance analysis (DSM-BIA). To examine the association between obesity and selected body composition indicators, Kruskal-Wallis ANOVA and Eta^2^ were used. The relationship between selected body composition indicators and percentile BMI zones was determined using the Kendall tau correlation.

**Results:**

In groups with different BMI percentile zones (normal weight, overweight, obese), ANOVA showed significant differences for girls and boys (*p* ˂.05) with high effect size (η^2^ ˂.26) in body weight, body fat mass index, body fat percentage, fat free mass index, fat-free mass percentage, visceral fat area, waist-to-hip ratio, waist circumference, protein mass and mineral mass. The highest degree of correlation among boys was between BMI values indicating overweight and obesity and fat free mass index and waist circumference, respectively (τ = .71, τ = .70, respectively). In girls, the highest correlation was found between classification of BMI percentile zones and waist circumference (t = .78).

**Conclusion:**

The characteristics of body composition are very useful determinants of health and nutrition status. Our data revealed a direct association between BMI value and chosen body composition indicators. The most accurate indicator of overweight and obesity in our study appears to be waist circumference for both male and female population.

## Background

Physical development can be considered as an indicator of the overall health status of the population. Obesity, defined as abnormal accumulation of fat in adipose tissue, has become the most common chronic and metabolic lifestyle disease. Obesity is a multifactorial disease [1] and over the past decades it has reached pandemic levels by doubling its worldwide prevalence [2]. Over the last few years, obesity has increased by 10–40 % in most European countries. Regardless of age, in Europe 10–25 % of people are obese, in America 20–25 %; however, in the case of women in Eastern Europe, the Mediterranean and some ethnic groups in the USA, up to 40 % of people are obese [3–5]. According to the National Anthropological Survey carried out in 2001, the current incidence of overweight and obesity in Slovak children and adolescents (7–18 years old) is as follows: 12.5 % among boys out of which 7.8 % were obese and 12.1 % among girls out of which 6.9 % were obese [6].

The prevalence of obesity among children and youths is alarming. The increase of prevalence of obesity is slower in the last few years than in past and achieved a plateau level in developed countries [7, 8]. Several authors consider adolescence to be a critical period for the development of obesity and the foundation of lifestyle diseases [9–11]. They also warn of increased weight among youths which is mainly determined by an increase in body fat. An increase in body mass index (BMI), incidence of metabolic and cardiovascular diseases in older age and an individual’s somatotype can be influenced, by as much as 30 %, by exogenous factors such as unhealthy dietary habits, lack of physical activity or sedentary behaviors [11].

The risk of serious chronic diseases depends on the degree of overweight/obesity and on the distribution of fat in the body. It is rather the distribution of body fat that plays a crucial role in metabolic processes related to obesity, not total body fat [12]. Obesity causes an increased risk of early onset of diabetes and cardiovascular disease even in young adults [13]. Sixty to eighty percent of obese adolescents carry obesity through to adulthood and have an 18-times greater risk of maintaining obesity and its complications [14]. Childhood obesity causes an increased risk of systolic and diastolic blood pressure [15]. Moreover, being in the top 90^th^ percentile causes a two times greater risk of hypertension (23 %) in comparison to children in the 75^th^ percentile (12 %); in the case of obesity, being in the top 95^th^ percentile, the risk is three times greater (34 %) than in children in the 75^th^ percentile [16].

At present, to assess the degree of obesity in children we use BMI percentile zones. BMI is significantly determined by several factors, such as age, gender, length of limbs and body composition [17, 18]. Although it is an important epidemiologic and clinical tool, BMI does not distinguish between fat mass and fat-free mass; thus individuals of the same BMI show varying levels of fatness [19, 20]. This is the reason why athletes can sometimes be included in overweight or obese groups; however, it is not only true of athletes as such examples can also be found in the general population. Conversely, an individual with a high proportion of fat mass and minimal amount of muscle mass is classified as being of normal weight. Nevertheless, BMI does not indicate fat localization in the body. Therefore, we believe that a more appropriate method for monitoring the prevalence of obesity is direct diagnostics of body composition indicators focused on fat (percentage body fat, fat mass index, visceral fat area, etc.) and active mass (fat free mass, fat free mass index, etc.). In relation to the body fat component, opinions found in available studies concerning its optimal percentage vary. Body fat levels ranging from 20 to 25 % in boys and 30 to 35 % in girls have been shown to be associated with health risk [21]. Childhood and adolescent overweight and obesity are important public health concerns [20].

The purpose of this study was to evaluate association between BMI percentile zones as health-risk for being overweight and obese and body composition indicators in high-school students.

In this study we would have attempted to answer the following questions: Are differences in body composition indicators among high school students classified by BMI percentile zones significant? What is the relationship between BMI percentile zones and selected body composition indicators?

## Methods

### Participants

A non-randomized cross-sectional study was used to describe selected indicators of body composition as predictors of health risk in high school students from the Prešov (Slovakia) region with respect to their gender differences.

The research group consisted of 1014 high school students aged between 14 and 21 years (boys *n* = 464; girls *n* = 550). Participants were selected using random stratified sampling. Strata were represented by individual districts of the Prešov region. In each of the strata, one school was selected by drawing lots. Overall, 14 high schools participated in the study; to be more specific four secondary grammar schools, four specialized schools (business academies, schools specializing in education and health care) and six secondary vocational schools. The same method of drawing lots was applied to select classes when one class was chosen from each grade, so that 56 classes were included in the research. Results can be generalized for the high school population in the Prešov region.

Due to verification of assumptions, the participants were divided into three sub-groups according to classification of the BMI percentile zones, namely into normal weight group (NW), overweight group (OW) and obese group (O) with respect to their gender.

Participants’ distribution into individual subgroups was carried out on the basis of the values of the Quetelet index (BMI) according to Slovak National Reference Standards based on the National Anthropological Survey 2001, which takes into account eating habits and local genotype [6]. BMI value below the 90^th^ percentile was assessed as normal weight, weight in the top 90^th^ percentile means being overweight and a value exceeding the 97^th^ percentile indicates obesity. The participants were assessed with regard to their age since the level of the degree of obesity shifts in relation to the participant’s age.

### Measures and procedures

Before analysis of body composition, the participants took part in basic measurements of anthropometric parameters. Body height was measured using a portable stadiometer (SECA 217, Hamburg, Germany) with an accuracy of 0.1 cm.

Body weight measured with an accuracy of 0.1 kg together with body composition was tested using direct segmental multi-frequency bioelectric impedance analysis (DSM-BIA). Bioelectrical impedance is a non-invasive, safe, fast and relatively cheap method for body composition analysis at the cellular level [22]. The method is based on measuring resistance and reactance of tissues. Resistance is determined by a tissue’s conductivity defined as a ratio of voltage and current. Reactance is defined as a tissue’s ability to slow down the current and cause a phase shift. This is an additional resistance that is dependent on the cell membranes’ capacity.

Bioimpedance was measured using an In Body 230 device (Biospace Co., Ltd.; Seoul, Korea). The In Body 230 device uses 8-point electrodes. Eight tactile electrodes are placed as follows: two of them are below each foot in anterior and posterior directions and two electrodes are in contact with the palm and thumb of each hand. The device works on the basis of ten repetitions of impedance measurement using two current frequencies, namely 20 and 100 kHz, in each of five body segments (right arm, left arm, trunk, right leg, left leg). The In Body 230 device shows high validity of results of directly measurable body composition indicators, i.e. fat mass, fat mass percentage and fat free mass, in comparison to Dual-energy X-ray absorptiometry (*r* = .94–.99) [23]. As stated in another study, bioelectric impedance showed excellent reliability with repeated measurements differing by less than .20 % with very small 95 % CI [24]. The device differentiates body weight into three components - total body water (intracellular and extracellular), dry mass (proteins and minerals) and body fat. Fat free mass contains a large amount of water and electrolytes and therefore it is a good conductor of electricity. Fat mass, which contains only a little water, is, on the other hand, a bad conductor [22].

The measurements were processed using Lookin’Body 3.0 version software (Biospace Co., Ltd.; Seoul, Korea).

Indirectly measurable parameters were calculated on the basis of software prediction equations for the given age category. Body composition was measured using the bioimpedance method under standard conditions described in bioimpedance analysis guidelines [25]. Room temperature was kept between 20 and 24 °C to prevent undesirable changes in body water composition [26]. In accordance with the manufacturer’s guidelines, the participants held out their arms and legs so that they would not come into contact with any other body segments during the procedure. If possible, the subjects were asked to avoid any vigorous physical activity for at least 24 h before the procedure, fast for 2 h and to urinate or defecate before the measurements.

The following parameters of body composition were monitored: percentage of body fat mass (PBF), body fat mass index (BFMI), percentage of fat free mass (PFFM), fat free mass index (FFMI), waist to hip ratio (WHR), waist circumference (WC), visceral fat area (VFA) and parameters of nutrition in the concrete protein mass (PM) and mineral mass (MM). The FFM and FM indexes are equivalent concepts to the BMI, and are defined as FFM/height^2^ (kg/m^2^) and FM/height^2^ (kg/m^2^), respectively. WHR, which is calculated based on the waist/hip circumference ratio, is used as an effective indicator of the Body Fat Mass [27]. Values of WC and WHR indicators were determined from the results of measurement on the InBody 230 device. The InBody uses its impedance index to provide a scientific estimation of the examinee’s value of circumference. However, validity and reliability of this estimation has not been published, yet.

### Statistical analysis

To describe the collected data, we used the mean as a measure of central tendency and standard deviation as a measure of variability. Supplementary data of basic characteristics also include minimal and maximal values.

The Shapiro-Wilk test was used to test normality of data distribution as a means of selection of statistical tests. To examine the association between obesity and selected body composition indicators, Kruskal-Wallis analysis of variance (K-W ANOVA) was used. To determine the significance of the difference among the BMI percentile zones, multiple comparisons for non-normally distributed variables (Mann-Whitney test with Bonferroni’s correction of *p*-value) were used.

The level of significance was set at 95 % for all statistical parameters (*p* <.05). Effect size (ES) of BMI percentile zones was determined using Eta squared values (*η* ^*2*^) from a formula for the non-parametric K-W ANOVA test (1):

1 $$ {\eta}^2=\left[\mathrm{H}/\left(\mathrm{n}-1\right)\right] $$

Effect size of the observed factors was assessed according to Thomas and Nelson [28], when *η* ^*2*^˂ .06 indicates a small effect, .06 ≤ *η* ^*2*^ ˂ .14 medium effect and *η* ^*2*^ ≥.14 large effect.

The relationship between selected body composition indicators and BMI percentile zones was determined using the Kendall Tau correlation based on the number of concordances and discordances in paired observations with statistical significance set at α = .01. The strength of the relationship was defined using Evans’s guide [29], where τ ˂.19 represents very weak, τ ˂.39 weak, τ ˂.59 moderate, τ ˂.79 strong and τ ≥.80 very strong association.

Statistical analysis was carried out using the Statistica v.12.0 program, (StatSoft, Inc.; Tulsa, USA).

## Results

The number of participants in each of the research subgroups, NW, OW and O divided according to BMI values, expressed also in percentage can be seen in Table 1. The participants’ average decimal age at the time of measurements was (mean ± standard deviation) 17.2 ± 1.3 years, average body height (BH) 170.3 ± 8.6 cm, average body weight (BW) was 62.9 ± 12.2 kg and average body mass index (BMI) was 21.6 ± 3.4 kg.m^−2^.

**Table 1:** The prevalence of being overweight and obese of participants according to BMI values

	Normal weight (NW)		Overweight (OW)		Obesity (O)	
	n	%	n	%	n	%
Male (*n* = 464)	364	78.4	43	9.3	57	12.3
Female (*n* = 550)	453	82.4	39	7.1	58	10.5
Overall (*n* = 1014)	817	80.6	82	8.1	115	11.3

Testing normality of data distribution using the Shapiro-Wilk test showed that several indicators of body composition in both male and female adolescents did not follow the Gaussian distribution (unpublished data).

Table 2 shows comprehensive values of the monitored body composition indicators of boys and their comparison from the perspective of BMI percentile zones. From this point of view, insignificant differences were found in parameters of age (H_2,463_ = 6.01; *p* = .521; *η* ^*2*^ = .012) and body height (H_2,463_ = .549; *p* = .760; *η* ^*2*^ = .001). Concerning body weight, significant differences between NW vs. OW and NW vs. O groups were detected (H_2,463_ = 207.4; *p* **<**.001; *η* ^*2*^ = .448), whilst between OW an O there was no significant difference. In parameters identifying the proportion of adipose tissue in participants’ bodies, significant differences in BFMI (H_2,463_ = 195.5; *p* **<**.001; *η* ^*2*^ = .422) and PBF (H_2,463_ = 162.1; *p* **<**.001; *η* ^*2*^ = .350) were discovered using Kruskal-Wallis analysis of variance and significant differences were determined between all three compared groups using multiple comparisons of mean. Participants’ inclusion in the overweight and obese groups was also demonstrated in VFA (H_2,463_ = 191.5; *p* **<**.001; *η* ^*2*^ = .414), when significant differences were found between individual compared pairs of data samples. Similar results of analysis of variance were also recorded in parameters assessing the proportion of active mass in participants’ bodies, namely in FFMI (H_2,463_ = 165.4; *p* **<**.001; *η* ^*2*^ = .357) and PFFM (H_2,463_ = 161.9; *p* **<**.001; *η* ^*2*^ = .350). In both cases, significantly lower values of active mass were found in the OW and O groups in comparison to the NW group. In the parameter of FFMI, there was no significant difference between the OW and O group recorded whilst in the PFFM parameter this difference was significant. Significant differences were also observed in parameters determined on the basis of circumference measures, i.e. WHR (H_2,463_ = 159.6; *p* **<**.001; *η* ^*2*^ = .345) and WC (H_2,463_ = 217.6; *p* **<**.001; *η* ^*2*^ = .470). In both cases, significant differences were found between NW vs. OW and NW vs. O. In comparison to OW and O groups, a significant difference was only detected in the WHR parameter. In parameters evaluating participants’ nutrition, significant changes were found between the groups with respect to classification of BMI percentile zones, as well (PM - H_2,463_ = 104.7; *p* **<**.001; *η* ^*2*^ = .226, MM - H_2,463_ = 115.6; *p* **<**.001; *η* ^*2*^ = .250). Along with increasing BMI, PM and MM also increased. Multiple comparisons of mean identified significant difference between NW vs. OW and NW vs. O; no differences in proportion of individual observed components were significant between OW vs. O.

**Table 2:** Values of body composition parameters in male adolescents and their comparison between the subgroups in relation to BMI percentile zones

Variables		*M*	*SD*	Min	Max	Multiple comparisons of mean
Age (years)	Overall	17.17	1.24	14.62	21.16	
	NW	17.09	1.22	14.62	21.16	
	OW	17.58	1.20	15.20	19.83	
	O	17.38	1.33	15.01	20.49	
BH (cm)	Overall	176.74	9.60	158.40	198.10	
	NW	176.64	6.60	158.40	198.10	
	OW	177.34	6.17	162.90	194.70	
	O	176.96	7.16	160.80	196.50	
BW (kg)	Overall	68.51 *^a^	11.98	45.10	119.90	
	NW	63.92	7.63	45.10	94.50	NW vs. OW (*p* ˂.01)
	OW	78.69	5.62	67.00	95.40	NW vs. O (*p* ˂.01)
	O	90.18	9.55	73.90	119.90	
BFMI (kg.m^-2^)	Overall	3.38 *^a^	2.15	.57	15.68	
	NW	2.56	.96	.57	5.91	NW vs. OW (*p* ˂.01)
	OW	4.49	1.33	2.30	7.56	NW vs. O (*p* ˂.01)
	O	7.83	2.32	3.56	15.68	OW vs. O (*p* ˂.05)
PBF (%)	Overall	14.64 *^a^	6.61	3.00	45.30	
	NW	12.32	3.95	3.00	26.90	NW vs. OW (*p* ˂.01)
	OW	17.92	5.32	9.40	30.70	NW vs. O (*p* ˂.01)
	O	26.92	6.52	13.30	45.30	OW vs. O (*p* ˂.01)
FFMI (kg.m^-2^)	Overall	18.52 *^a^	1.88	13.88	23.80	
	NW	17.90	1.49	13.88	21.58	NW vs. OW (*p* ˂.01)
	OW	20.51	1.34	17.08	22.53	NW vs. O (*p* ˂.01)
	O	20.93	1.41	18.08	23.80	
PFFM (%)	Overall	85.36 *^a^	6.61	54.73	96.99	
	NW	87.67	3.95	73.09	96.99	NW vs. OW (*p* ˂.01)
	OW	82.07	5.31	69.32	90.53	NW vs. O (*p* ˂.01)
	O	73.09	6.52	54.73	86.73	OW vs. O (*p* ˂.01)
WHR	Overall	.83 *^a^	.06	.74	1.50	
	NW	.81	.04	.74	.95	NW vs. OW (*p* ˂.01)
	OW	.86	.05	.78	.97	NW vs. O (*p* ˂.01)
	O	.93	.05	.82	1.50	OW vs. O (*p* ˂.01)
WC (cm)	Overall	79.11 *^a^	9.74	65.90	116.50	
	NW	75.11	4.87	65.90	92.90	NW vs. OW (*p* ˂.01)
	OW	86.35	4.83	77.50	96.20	NW vs. O (*p* ˂.01)
	O	99.19	7.64	86.00	116.50	
VFA (cm^2^)	Overall	46.21 *^a^	31.20	5.00	187.20	
	NW	34.46	18.27	5.00	88.50	NW vs. OW (*p* ˂.01)
	OW	66.16	20.69	29.80	112.50	NW vs. O (*p* ˂.01)
	O	106.22	26.32	58.10	187.20	OW vs. O (*p* ˂.01)
PM (kg)	Overall	11.50 *^a^	1.54	7.20	16.60	
	NW	11.10	1.33	7.20	15.80	NW vs. OW (*p* ˂.01)
	OW	12.86	1.31	10.40	16.50	NW vs. O (*p* ˂.01)
	O	13.04	1.46	10.10	16.60	
MM (kg)	Overall	3.95 *^a^	.57	2.57	6.60	
	NW	3.80	.48	2.57	5.80	NW vs. OW (*p* ˂.01)
	OW	4.44	.48	3.35	5.81	NW vs. O (*p* ˂.01)
	O	4.58	.56	3.50	6.60	

*Note. BH* body height, *BW* body weight, *BFMI* body fat mass index, *PBF* percentage of body fat, *FFMI* fat free mass index, *PFFM* percentage of fat free mass, *WHR* waist to hip ratio, *WC* waist circumference, *VFA* visceral fat area, *PM* protein mass, *MM* mineral mass, *NW* normal weight group, *OW* overweight group, *O* obesity group, * *p* ˂.05 Kruskal-Wallis ANOVA, ^a^ large effect size

Table 3 presents values of selected body composition indicators as predictors of girls’ health with regard to classification of BMI percentile zones. As results indicate, no significant difference between the groups was found in terms of decimal age (H_2,549_ = 2.95; *p* = .229; *η* ^*2*^ = .005) and body height (H_2,549_ = 2,10; *p* = .351; *η* ^*2*^ = .004). However, a significant difference was revealed in body weight (H_2,549_ = 215.5; *p* <.001; *η* ^*2*^ = 393), which, using multiple comparisons of mean, was identified between NW vs. OW and NW vs. O. No significant difference was detected between the groups of overweight and obese female adolescents. Kruskal-Wallis analysis of variance revealed significant differences between participants with respect to classification of BMI percentile zones in all observed indicators of body composition characterizing proportion of adipose tissue: BFMI (H_2,549_ = 225.6; *p* <.001; *η* ^*2*^ = .411), PBF (H_2,549_ = 196.7; *p* <.001; *η* ^*2*^ = .358), VFA (H_2,549_ = 188.8; *p* <.001; *η* ^*2*^ = .344), proportion of fat free mass: FFMI (H_2,549_ = 155,6; *p* <.001; *η* ^*2*^ = .283), PFFM (H_2,549_ = 196.5; *p* <.001; *η* ^*2*^ = .358), abdominal obesity parameters: WHR (H_2,549_ = 198.2; *p* <.001; *η* ^*2*^ = .361), WC (H_2,549_ = 227.7; *p* <.001; *η* ^*2*^ = .415) and nutrition parameters: PM (H_2,549_ = 94.2; *p* <.001; *η* ^*2*^ = .172) and MM (H_2,549_ = 103.4; *p* <.001; *η* ^*2*^ = .188). Multiple comparisons of mean indicated significant differences between NW vs. OW and NW vs. O in all monitored parameters. Significant differences between OW vs. O were only found in the FFMI parameter, whilst in other parameters there were no significant differences between these groups.

**Table 3:** Values of body composition parameters in female adolescents and their comparison between the subgroups in relation to BMI percentile zones

Variables		*M*	*SD*	Min	Max	Multiple comparisons of mean
Age (years)	Overall	17.24	1.24	14.81	21.19	
	NW	17.22	1.24	14.81	21.19	
	OW	17.49	1.29	15.22	19.33	
	O	17.26	1.28	15.22	20.21	
BH (cm)	Overall	164.9	5.82	147.00	181.60	
	NW	164.72	5.73	147.00	181.60	
	OW	165.65	6.52	153.30	179.10	
	O	165.54	5.98	150.90	177.50	
BW (kg)	Overall	58.16 *^a^	10.20	37.20	100.20	
	NW	54.74	6.69	37.20	70.60	NW vs. OW (*p* ˂.01)
	OW	68.66	5.80	58.70	80.40	NW vs. O (*p* ˂.01)
	O	77.77	7.98	61.00	100.20	
BFMI (kg.m^-2^)	Overall	5.91 *^a^	2.46	1.22	16.3	
	NW	5.50	1.52	1.22	10.0	NW vs. OW (*p* ˂.01)
	OW	8.59	1.90	6.44	10.90	NW vs. O (*p* ˂.01)
	O	10.86	1.94	6.62	16.30	
PBF (%)	Overall	26.81 *^a^	7.10	8.30	48.00	
	NW	24.67	5.47	8.30	42.10	NW vs. OW (*p* ˂.01)
	OW	34.38	4.13	26.00	43.00	NW vs. O (*p* ˂.01)
	O	38.08	4.36	25.40	48.00	
FFMI (kg.m^-2^)	Overall	15.48 *^a^	1.34	12.27	20.07	
	NW	15.11	1.90	12.27	18.24	NW vs. OW (*p* ˂.01)
	OW	16.39	1.10	14.47	18.64	NW vs. O (*p* ˂.01)
	O	17.50	1.10	15.66	20.07	OW vs. O (*p* ˂.01)
PFFM (%)	Overall	73.34 *^a^	7.10	52.01	91.73	
	NW	75.45	5.47	57.94	91.73	NW vs. OW (*p* ˂.01)
	OW	65.62	4.14	57.03	74.02	NW vs. O (*p* ˂.01)
	O	61.93	4.36	52.01	74.66	
WHR	Overall	.85 *^a^	.05	.73	1.60	
	NW	.83	.04	.73	.96	NW vs. OW (*p* ˂.01)
	OW	.91	.03	.83	.97	NW vs. O (*p* ˂.01)
	O	.94	.05	.85	1.60	
WC (cm)	Overall	78.17 *^a^	9.26	60.80	119.20	
	NW	74.85	5.63	60.80	92.20	NW vs. OW (*p* ˂.01)
	OW	88.55	4.30	79.80	96.30	NW vs. O (*p* ˂.01)
	O	96.64	7.20	84.90	119.20	
VFA (cm^2^)	Overall	62.85 *^a^	31.17	5.00	184.70	
	NW	53.2	22.53	5.00	119.90	NW vs. OW (*p* ˂.01)
	OW	96.23	21.71	42.10	129.00	NW vs. O (*p* ˂.01)
	O	115.30	25.86	54.50	184.70	
PM (kg)	Overall	8.26 *^a^	1.00	6.10	12.50	
	NW	8.50	.86	6.10	10.90	NW vs. OW (*p* ˂.01)
	OW	8.86	.98	6.80	10.90	NW vs. O (*p* ˂.01)
	O	9.46	1.40	7.20	12.50	
MM (kg)	Overall	3.01 *^a^	.38	2.17	4.63	
	NW	2.93	.32	2.17	3.80	NW vs. OW (*p* ˂.01)
	OW	3.27	.37	2.58	4.60	NW vs. O (*p* ˂.01)
	O	3.49	.40	2.49	4.63	

Note: *BH* body height, *BW* body weight, *BFMI* body fat mass index, *PBF* percentage of body fat, *FFMI* fat free mass index, *PFFM* percentage of fat free mass, *WHR* waist to hip ratio, *WC* waist circumference, *VFA* visceral fat area, *PM* protein mass, *MM* mineral mass, *NW* normal weight group, *OW* overweight group, *O* obesity group, * *p* ˂.05 Kruskal-Wallis ANOVA, ^a^ large effect size

**Fig. 1:**
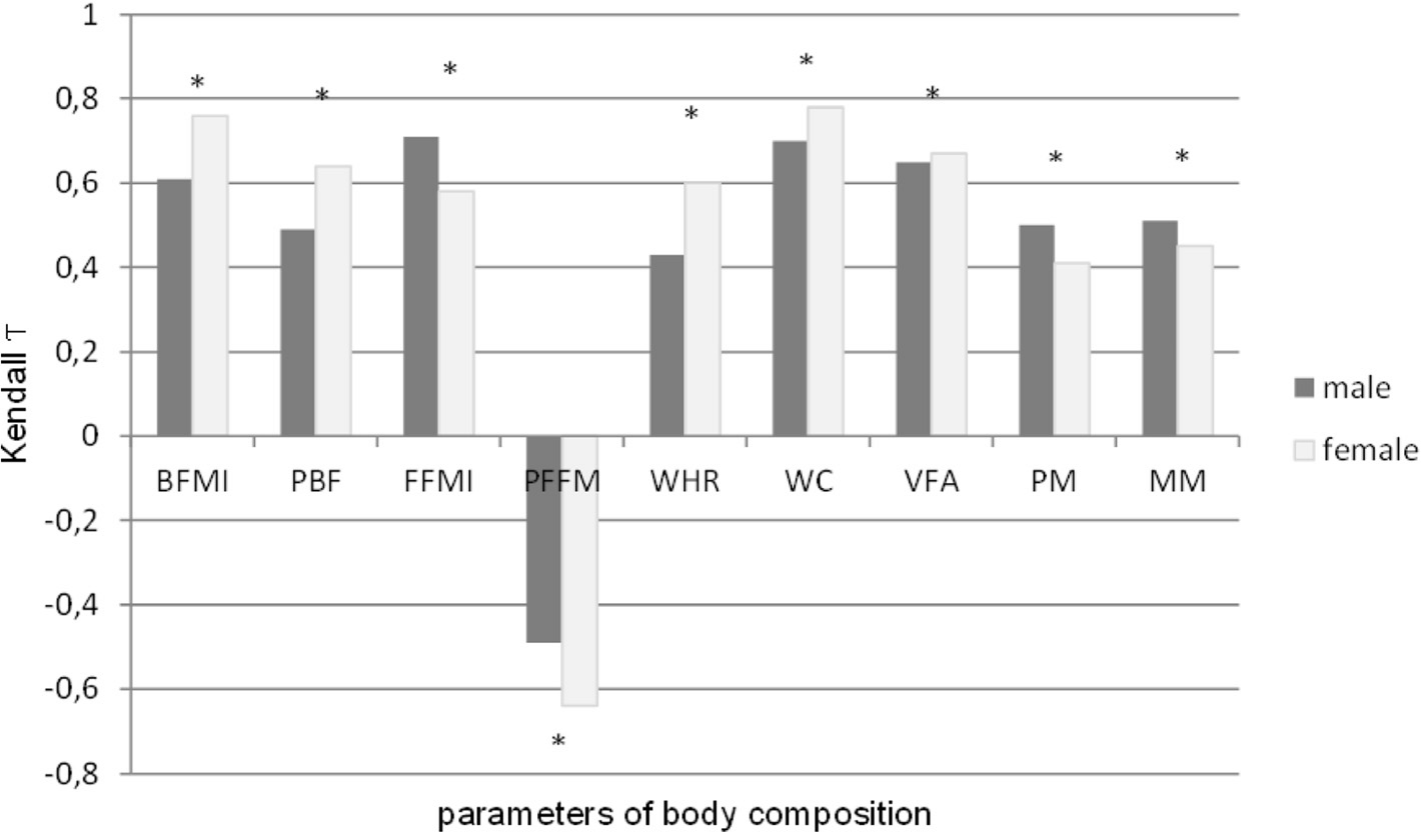
Kendall tau correlations between body mass index and chosen indicators of body composition according to gender (* significance *p* ˂.05)

Correlations were calculated between BMI percentile zones and individual observed indicators of body composition with respect to gender (Fig. 1). In male adolescents, from the perspective of the major factor, i.e. classification of BMI percentile zones, positive moderate correlation between the following body composition parameters was found: PBF (τ = .49), WHR (τ = .43), PM (τ = .50) and MM (τ = .51). Negative moderate correlation was determined between the major factor and PFFM (τ = −.49). Positive strong correlation was calculated between classification of BMI percentile zones and BFMI (τ = .61) and VFA (τ = .65). The strongest correlation was detected in relation to WC or FFMI, respectively (τ = .70, τ = .71, respectively). In the case of female adolescents, results of Kendall’s correlation analysis were similar; positive moderate correlation was identified between BMI classification and nutrition parameters PM (τ = .41) and MM (τ = .45) as well as FFMI (τ = .58). Regarding the PFFM parameter, negative strong correlation was found (τ = −.64). Positive strong correlation was calculated in PBF (τ = .64), WHR (τ = .60) and VFA (τ = .67). The highest level of association, approaching very strong positive correlation, was observed in relation to BFMI (τ = .76) and WC (τ = .78).

Results of correlation analysis were also confirmed by the significance of correlation at the probability level (*p* <.05). Results of correlation analysis are demonstrated in the graph in Fig. 1.

## Discussion

Currently, most of the pediatric literature relies on BMI to identify children as overweight or obese. BMI may be useful in screening but the standard errors of the predictions were about 3 kg for total body fat and 4 % for percent body fat [30]. Therefore, it is not a useful predictor of body fatness. From the point of view of assessing obesity and being overweight using BMI standards, 8.1 % of our sample was overweight and 11.3 % was obese. In the group of male participants, it was 9.3 % or 12.3 %, respectively, and in the group of female participants 7.1 % or 10.5 %, respectively. We have confirmed the fact that a lower number of overweight and obese individuals was to be found among girls in comparison to boys, which is consistent with the results of the National Anthropological Survey 2001 [6]. A similar state in adolescent population was according to Bibiloni et al. [31] also found in other European countries.

The results of a previous study demonstrate a strong relationship between chronic disease risk factors and percent fat in children and youths that varies by age in boys and girls [21]. Concerning the group of boys, in the parameters describing proportion of adipose tissue in the body we recorded significant differences in BFMI and PBF in all three monitored groups. The recorded median value for the group of obese participants was 26.9 %. Williams and colleagues identified PBF threshold of 25 % in boys, which means an increased health risk [32]. The groups of participants with normal weight or overweight showed PBF values below the indicated threshold (Table 2). Participants’ inclusion in the overweight and obese groups was also demonstrated in VFA when significant differences were found between individual compared pairs of data samples.

In the girl’s group, significant differences were revealed in all observed indicators of body composition characterizing proportion of adipose tissue in participants’ bodies, namely in BFMI, PBF and VFA. The average value of PBF was 26.8 %. In another study, median PBF at age 18 for girls was 27.8 %. By the age of 18, girls had approximately 1.5 times greater PBF than boys [21]. These findings are very similar to our results when 18-year-old girls achieved a value of 27.1 and it was 1.7-times higher than in boys of the same age. Percent fat is significantly related to risk factor levels [32]. There has been increasing scientific interest in fat mass largely because of its relationship to health status [33]. Using a modified Slaughter equation, Williams and colleagues identified PBF thresholds 30 % in girls and 25 % in boys that were indicative of an increased risk of being in the highest quintile for blood pressure and serum lipoproteins in adolescents [32]. With respect to these results, some girls and boys from our sample appear to be at increased risk. Such participants were detected throughout the whole range of BMI percentile zones. In the case of girls, 31.3 % were found to be at the risk zone, out of which 18.3 % were from NW group, 84.6 % from OW group and 98.3 % from O group. Regarding boys, 9 % of them were at the risk level. Their proportion among NW, OW and O groups was 0.5, 9.3, 62 %, respectively.

With respect to this fact, it would be suitable to create standards for individual categories – cut off score – for all observed parameters according to which the assessment of results would be more exact. PBF thresholds of 22.3 and 35.1 % in boys and 31.4 and 38.6 % in girls (at age 18 years) were found to be indicative of “low” and “high” metabolic syndrome risk [17]. The study identifies thresholds (at age 18.0–18.9 years) of 22.3 and 35.1 % in boys which approximate the cutoffs for BMI of 25 and 35 kg/m^2^ in the adult study. In girls, the identified thresholds of 31.4 and 38.6 % correspond to the cut offs for BMI of 25 and 30 kg/m^2^ in the adult study. In general, it appears that perhaps the girls’ threshold could be adjusted to a higher percentile to allow it to more closely align to the threshold of 35 kg/m^2^ (instead of 30 kg/m^2^) [33]. Although the approach of Zhu and colleagues [34] incorporated BMI to define healthy PBF standards, rather than PBF alone, the odds of metabolic syndrome drastically escalate as adult BMI increases above 25 and 30 kg/m^2^ [35]. Furthermore, childhood overweight and obesity using these BMI thresholds is predictive of adult obesity [36]. The recent trends in the prevalence of childhood obesity and its associated health risks are well documented [37].

In screening programs, the use of simple anthropometric indices (i.e. BMI, skinfold thicknesses and waist circumference) has become popular to identify children and adolescents who are overweight / obese or at risk of developing excess adiposity [38].

According to standards for WC set by the WHO [2], which suggest that the risk for men is over the value of 94 and 80 cm for women, respectively. In our research group, male participants from the obese group took place in the risk zone (M = 99.2 cm), with respect to the median value, and in female participants it was the overweight group (M = 88.5 cm) and obese group (M = 96.6 cm). As the above mentioned results indicate, WC is an indicator using which it is easy to identify individuals at risk of being overweight and obese. Concerning WC, several studies confirmed a high correlation with BMI in children and adolescents [39, 40]. Considering the strong correlation between these two parameters, it may be inferred that they have no independent effect.

Pereira et al. observed that the covariance between them is reduced, and thus the combined use of BMI and WC would be a better predictor of health risk for children and adolescents [41]. As stated by Ochiai et al., it is essential to consider gender and obesity when using BMI as a surrogate for %BF and WC for epidemiological use [42]. Similarly, another study revealed a high correlation between WC and PBF in adolescents aged between 12 and 18 years (*r* = .85) [39]. PBF is considered as the most accurate predictor of cardiovascular diseases.

Based on results of the study by Laurson et al., body fat was highly accurate at detecting elevated WC, although this is to be expected since both are assessments of adiposity and are generally highly correlated [43]. This could indicate that WC alone could be used to identify youths at risk of metabolic syndrome, which is intuitive since WC is an integral component of the syndrome [43]. Gluteal adipocyte weight is more highly correlated with total body fat than adipocyte weight at other sites [29]. Recent studies in obese young people have shown that VFA tends to explain greater proportions of the variance in cardiovascular disease (CVD) risk factors than other measures of adiposity such as total percent body fat, total body fat mass, waist-to-hip ratio or subcutaneous abdominal adipose tissue. The waist-to-hip ratio was a relatively strong predictor of total cholesterol, total cholesterol/HDLC, LDLC, and Apo B [44].

In the screened sample, the relationship between VFA, WC and WHR was revealed in individual age categories, which confirms the above mentioned findings. In male participants, from the perspective of the major factor, i.e. BMI percentile zones, positive moderate correlation between the parameters of PBF and WHR was found. Positive strong correlation was calculated between classification of BMI percentile zones and BFMI and VFA and the highest correlation was detected in relation to WC. It was similar in girls when positive strong correlation was calculated for PBF, WHR and VFA. The highest measure of association, approximating very strong positive correlation, was observed in relation to BFMI and WC indicators. Circumference measurement is influenced by tissues (adipose tissue, muscle and bone) that do not correlate highly. Therefore, ratios between circumferences may lack specificity regarding adipose tissue distribution. This has been demonstrated using hospital records from computed tomography [30]. It was shown that the ratio of abdominal to hip cross-sectional areas correlated strongly with intra-abdominal fat but also with muscle and bone areas [30].

In the indicator describing proportion of active mass with respect to subject’s height, FFMI, we recorded an increasing value in all monitored groups of both male and female adolescents. The highest values were achieved by participants classified as O and, on the contrary, the lowest values were found among participants classified as NW. These results are in accordance with findings by Sabino et al. [45]. The opposite result was recorded in the relative value of fat free mass (PFFM) where with increasing BMI the values of PFFM decreased. Median value of FFMI in male participants was lower than reference values reported by Schutz et al. [46] and median values of all subgroups were in the range of 5–95^th^ percentile. Concerning female participants, median value of FFMI was in affinity with the reference value mentioned by [46].

An important limitation of the study is that the participants could not be randomly selected. The results are thus only valid for adolescent population in the Prešov region, Slovakia, and can be generalized with certain limits. Furthermore, representation of obese subjects was low; on the other hand, its percentage corresponds with the incidence of obesity in the Slovak Republic. The use of the described method of the analysis of body composition is in pediatric practice partially limited due to the device’s purchase cost. However, as indicated by the results of correlation analysis, the best predictor of overweight and obesity appears to be waist circumference which is, moreover, inexpensive when using the tape measure. On the other hand, it is necessary that the person performing such measurement has to be properly trained in terms of the conditions under which the measurement is taken, taking into account location and time of the measurement, food intake or abdominal muscle tonus during the measurement.

## Conclusions

The biggest association was determined in relation to waist circumference and fat free mass index. Since FFMI significantly depends on body height, based on these results we can consider WC the best predictor of the BMI percentile zones in boys of this age category. In girls, the highest association was found in relation to body fat mass index and waist circumference. Similarly as in boys, the WC parameter appears as the best predictor of the BMI percentile zones in girls. Moreover, based on the results of multiple comparisons, similar values of body composition indicators between overweight and obese groups classified according to the BMI percentile zones were observed.

Despite the fact that BMI is an indicative parameter only and does not capture differences in body composition, it matches relatively well with distribution of our participants with respect to fat related parameters, namely BFMI, WHR and WC, especially in boys. Concerning our female participants, the results are not so clear as BMI appears to be only little sensitive, especially when related to PBF.

The importance of such studies lies in the fact that, based on their results, it is possible to identify specificities of individual regions; conversely, they reflect local conditions and habits which may differ across regions. It seems that it is necessary to create national standards for each age category in individual body composition indicators which realistically reflect endogenous and exogenous factors affecting the body. Future studies with large, representative samples are needed to precisely define the percent fat risk factor and BMI relationships at different ages.

## Abbreviations

BFMI: Body fat mass index
BH: Body height
BMI: Quetelet index (body mass index)
BW: Body weight
DSM-BIA: Direct segmental multi-frequency bioelectric impedance analysis
FFMI: Fat free mass index
K-W ANOVA: Kruskal-Wallis analysis of variance
MM: Mineral mass
NW: Normal weight group
O: Obese group
OW: Overweight group
PBF: Percentage of body fat mass
PFFM: Percentage of fat free mass
PM: Protein mass
VFA: Visceral fat area
WC: Waist circumference
WHR: Waist to hip ratio
*η*^*2*^: Effect size - Eta squared
τ: Kendall Tau correlation
